# Augmented Reality and Intraoperative Navigation in Sinonasal Malignancies: A Preclinical Study

**DOI:** 10.3389/fonc.2021.723509

**Published:** 2021-11-01

**Authors:** Axel Sahovaler, Harley H. L. Chan, Tommaso Gualtieri, Michael Daly, Marco Ferrari, Claire Vannelli, Donovan Eu, Mirko Manojlovic-Kolarski, Susannah Orzell, Stefano Taboni, John R. de Almeida, David P. Goldstein, Alberto Deganello, Piero Nicolai, Ralph W. Gilbert, Jonathan C. Irish

**Affiliations:** ^1^ Department of Otolaryngology–Head and Neck Surgery/Surgical Oncology, Princess Margaret Cancer Centre/University Health Network, Toronto, ON, Canada; ^2^ Guided Therapeutics (GTx) Program, Techna Institute, University Health Network, Toronto, ON, Canada; ^3^ Unit of Otorhinolaryngology–Head and Neck Surgery, University of Brescia–ASST “Spedali Civili di Brescia, Brescia, Italy; ^4^ Section of Otorhinolaryngology–Head and Neck Surgery, University of Padua–Azienda Ospedaliera di Padova, Padua, Italy

**Keywords:** augmented reality, intraoperative navigation, surgical margins, sinonasal tumors, surgical margin delineation

## Abstract

**Objective:**

To report the first use of a novel projected augmented reality (AR) system in open sinonasal tumor resections in preclinical models and to compare the AR approach with an advanced intraoperative navigation (IN) system.

**Methods:**

Four tumor models were created. Five head and neck surgeons participated in the study performing virtual osteotomies. Unguided, AR, IN, and AR + IN simulations were performed. Statistical comparisons between approaches were obtained. Intratumoral cut rate was the main outcome. The groups were also compared in terms of percentage of intratumoral, close, adequate, and excessive distances from the tumor. Information on a wearable gaze tracker headset and NASA Task Load Index questionnaire results were analyzed as well.

**Results:**

A total of 335 cuts were simulated. Intratumoral cuts were observed in 20.7%, 9.4%, 1.2,% and 0% of the unguided, AR, IN, and AR + IN simulations, respectively (p < 0.0001). The AR was superior than the unguided approach in univariate and multivariate models. The percentage of time looking at the screen during the procedures was 55.5% for the unguided approaches and 0%, 78.5%, and 61.8% in AR, IN, and AR + IN, respectively (p < 0.001). The combined approach significantly reduced the screen time compared with the IN procedure alone.

**Conclusion:**

We reported the use of a novel AR system for oncological resections in open sinonasal approaches, with improved margin delineation compared with unguided techniques. AR improved the gaze-toggling drawback of IN. Further refinements of the AR system are needed before translating our experience to clinical practice.

## Introduction

The complex anatomy and close proximity of critical structures in the sinonasal region represent a major challenge for surgeons when treating advanced tumors in this location, and incomplete resections are not uncommon, both in open and endoscopic approaches (1, 2). Intraoperative navigation (IN) has been proposed as a potential strategy to improve surgical margins (3). IN enables co-registration of computed tomography (CT) and/or magnetic resonance imaging (MRI) studies with surgical instruments. As a result, real-time feedback of instrument location is provided in order to help the surgeon during the operation.

Our group has recently published an advanced IN system for open sinonasal approaches during the resection of locally aggressive cancers (4). This technology not only allows the surgeon to locate a registered instrument or pointer tool in two dimensions but also introduces planar cutting tool capabilities along with three-dimensional (3D) volume rendering. Therefore, the surgeon can anticipate the direction of the cutting instrument in 3D planes with respect to the tumor and improve accuracy of margin delineation. Still, one key drawback of all IN systems is that the information is displayed outside the surgical field, and therefore surgeons are forced to switch their gaze between the actual procedure and the navigation monitor, which can impact safety and efficiency.

Augmented reality (AR) uses visual inputs to enhance the user’s natural vision and therefore can integrate navigation information onto the surgical field (5). This feature can potentially address the gaze-toggling drawback of IN and at the same time provide valuable information to the surgeon, for example, facilitating tumor localization and delineation. Reports of AR in otolaryngology–head and neck surgery are scarce, and most of them come from endoscopic sinus surgery (6), transoral robotic surgery (7), and otology (8, 9). Nevertheless, open sinonasal procedures also represent an adequate indication for AR. The rigid structure of the sinonasal region facilitates the co-registration processes required for AR, and the high rates of incomplete resections in advanced sinonasal tumors could be improved with the use of this technology.

The objective of this study was to report the first use of a novel AR system in open sinonasal tumor resections in preclinical models and to compare an AR approach with an advanced IN navigation system.

## Materials and Methods

### Tumor Models

**Figure 1:**
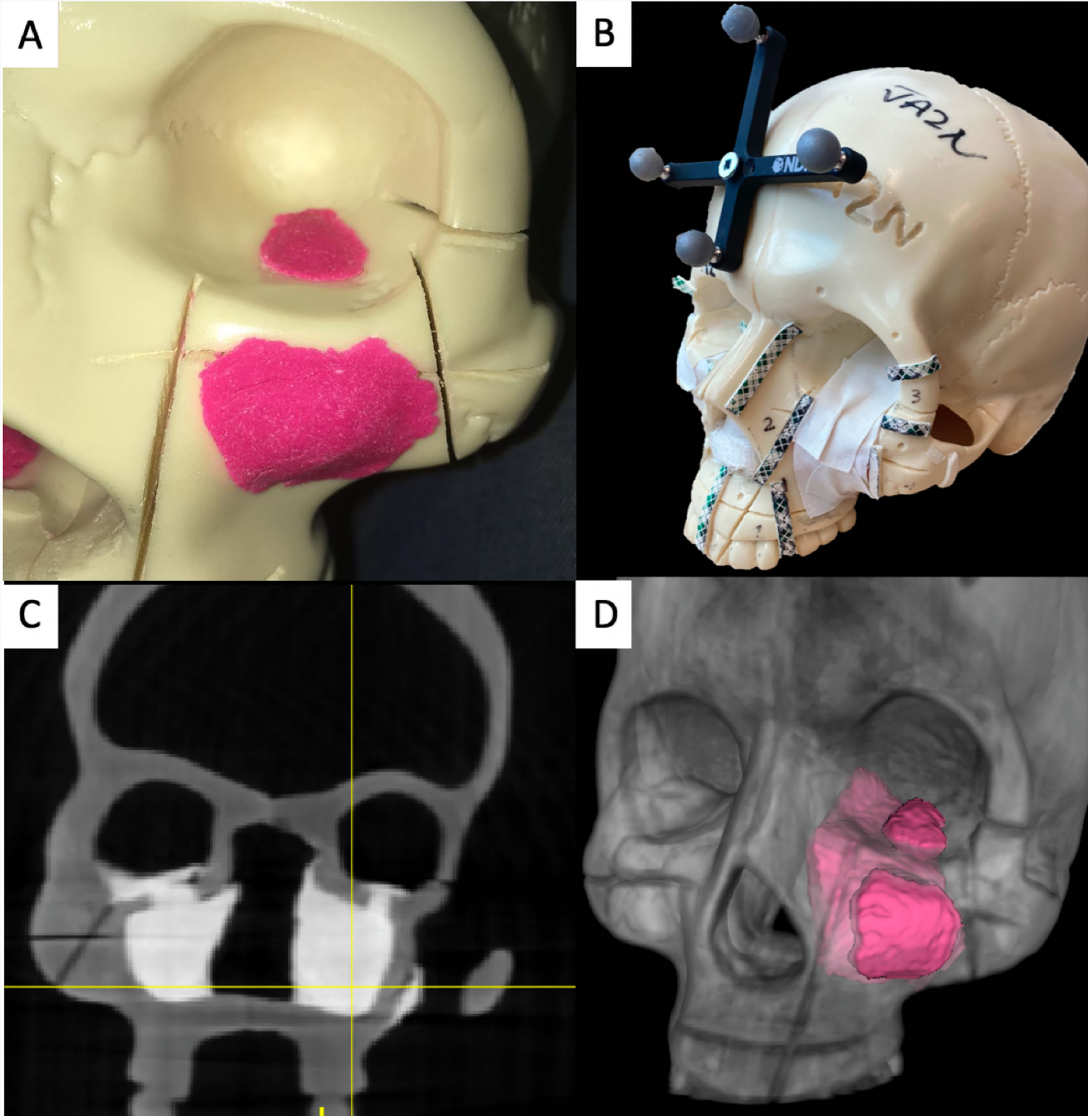
Tumor models, image acquisition, and tumor contouring. **(A)** Artificial skulls with moldable material simulating advanced sinonasal tumors. **(B)** Final tumor model, with the tumors covered with white tape. The areas to be osteotomized are delineated with visible tape and marked with numbers. A four-sphere reference tool drilled to the skull to co-register the intraoperative navigation. **(C)** Higher attenuation of the artificial tumor models in the cone-beam computed tomography (CBCT) images. **(D)** Three-dimensional (3D) contouring of the left tumor.

Two artificial skulls (Sawbones^®^) and a moldable material (Play-Doh^®^) mixed with acrylic glue were employed to build four locally advanced sinonasal tumor models (**Figure 1A**). Tumor surfaces were disguised with tape. Five different areas to be osteotomized were delineated: palatal osteotomy (Pa), fronto-maxillary junction (FMJ), latero-inferior orbital rim (LIOR), zygomatic arch (Zy), and pterygomaxillary junction (PMJ) (**Figure 1B**).

### Image Acquisition and Tumor Contouring

Cone-beam computed tomography (CBCT) scans acquired 3D images of the skull models (9). Tumors showed higher x-ray attenuation than the artificial bone (**Figure 1C**). Tumor contouring was performed semiautomatically (10). First, a global threshold was applied to provide a quick, coarse segmentation, and then manual refinement was used to smooth the segmentation (**Figure 1D**).

### Advanced Intraoperative Navigation System

**Figure 2:**
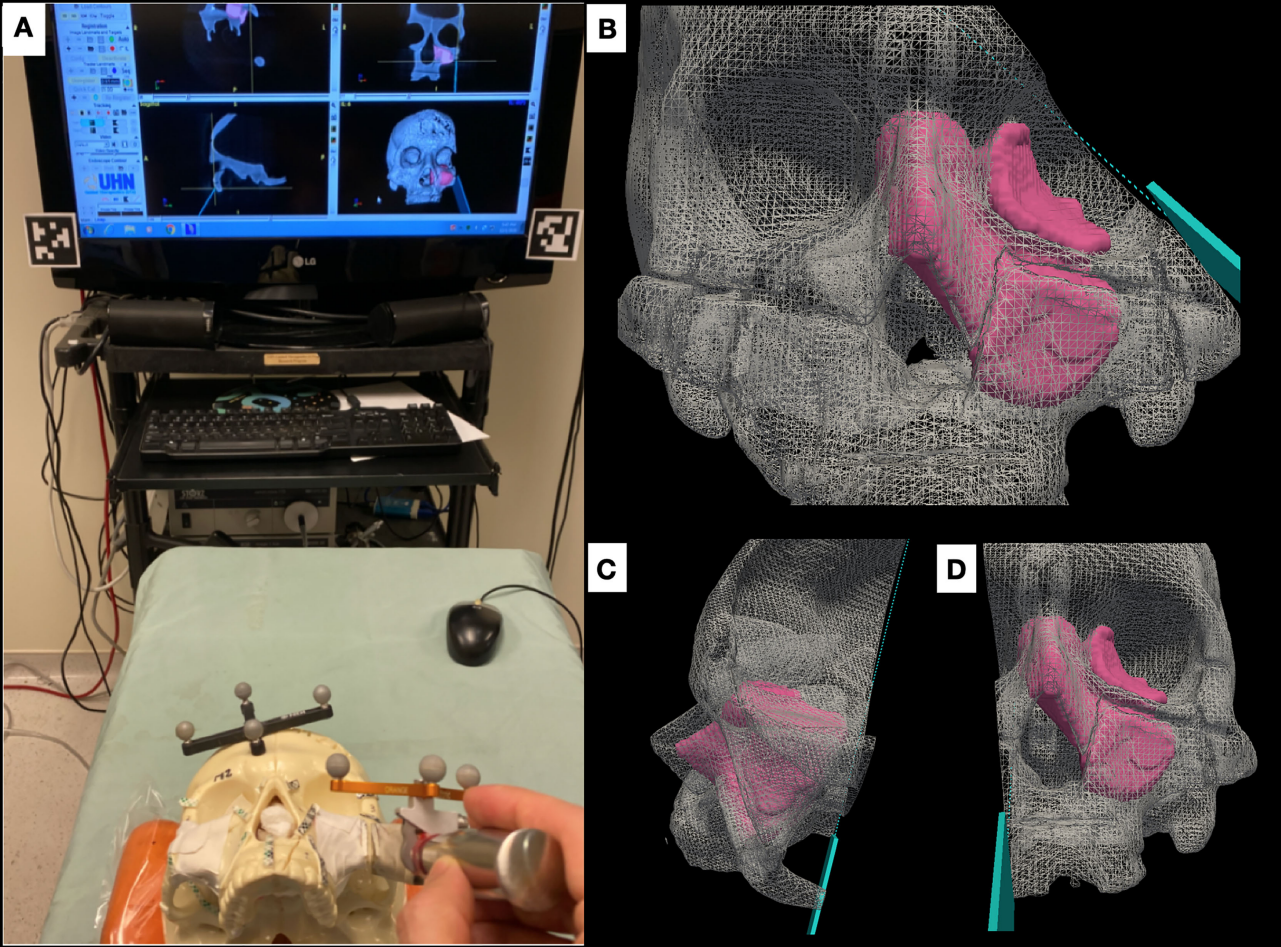
Advanced intraoperative navigation system. **(A)** Setup of the system, depicting the triplanar cutting views and the three-dimensional (3D) rendering of the tumor on the screen. The skull and the cutting instrument (in this case an osteotome) are referenced to be tracked and co-registered. **(B–D)** The system allows users to visualize cutting trajectories of the instrument with respect to the tumor. **(B)** Cutting of the latero-inferior orbital rim, **(C)** pterygomaxillary junction, and **(D)** palate.

An in-house navigation software package, GTx-Eyes, processed and displayed the CBCT images (11). This software has been proven useful in a breadth of surgical oncology subspecialties (12–15), and the technical aspects are described elsewhere (16). Tumor and margin segmentations were superimposed on triplanar views and also shown as 3D surface renderings (**Figure 2A**). Tool tracking was achieved by a stereoscopic infrared camera. Image-to-tracker registration was obtained by paired-point matching of predrilled divots in the skull by means of a tracked pointer. A four-sphere reference tool was drilled to the skull. A fiducial registration error of ≤1 mm was deemed acceptable. A three-sphere reference was attached to an osteotome and calibrated. This advanced IN system allows visualization of the entire trajectory of the cutting instrument with respect to the tumor in 3D views (**Figures 2B–D**, **Supplementary Video**).

### Augmented Reality System

**Figure 3:**
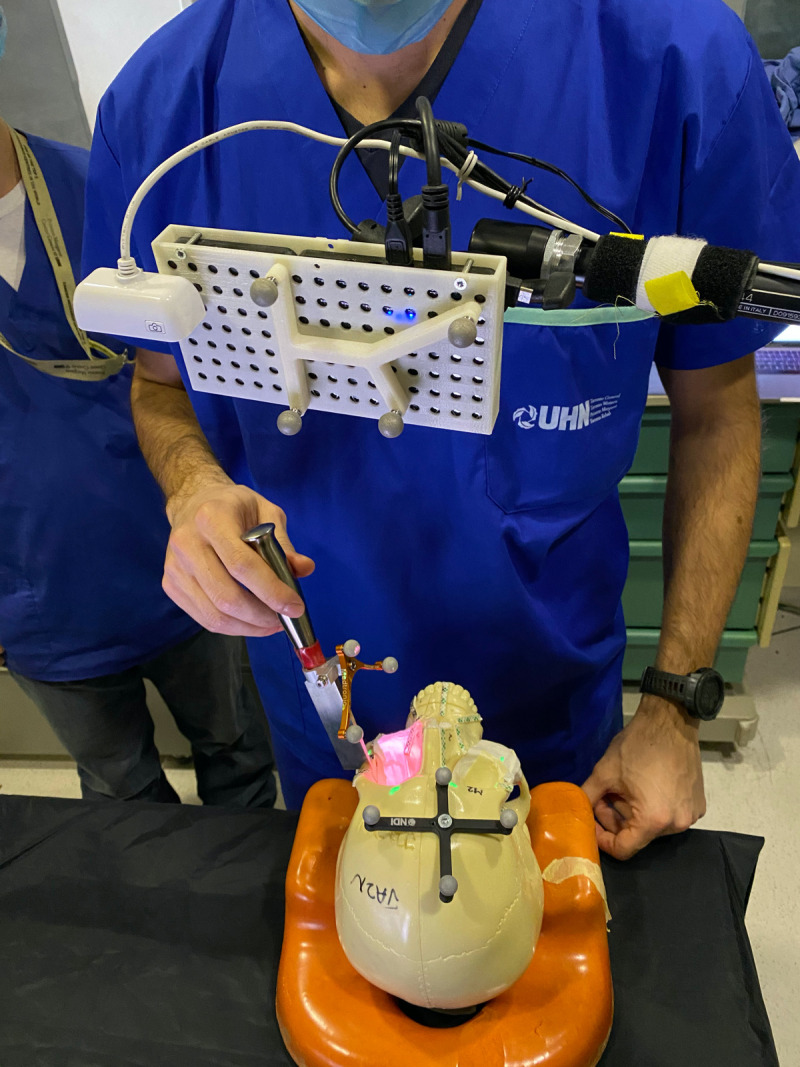
Augmented reality system mounted and projecting.

The AR system was composed of a portable high-definition projector (PicoPro, Celluon Inc., Federal Way, WA, USA), a stereoscopic infrared camera (Polaris Spectra, NDI, Waterloo, ON, Canada), a USB 2.0M pixel generic camera (ICAN Webcam 2MP, China), and a laptop computer (M4500, Precision laptop, Dell, Round Rock, TX, USA). A custom-made 3D printed case was fabricated to anchor a four-sphere reference tool and contain the other elements of the AR system (**Figure 3**). The technical details of this system including the kinematic transformation, reference system, and conventional image-to-tracker registration method have also been described elsewhere (17).

**Figure 4:**
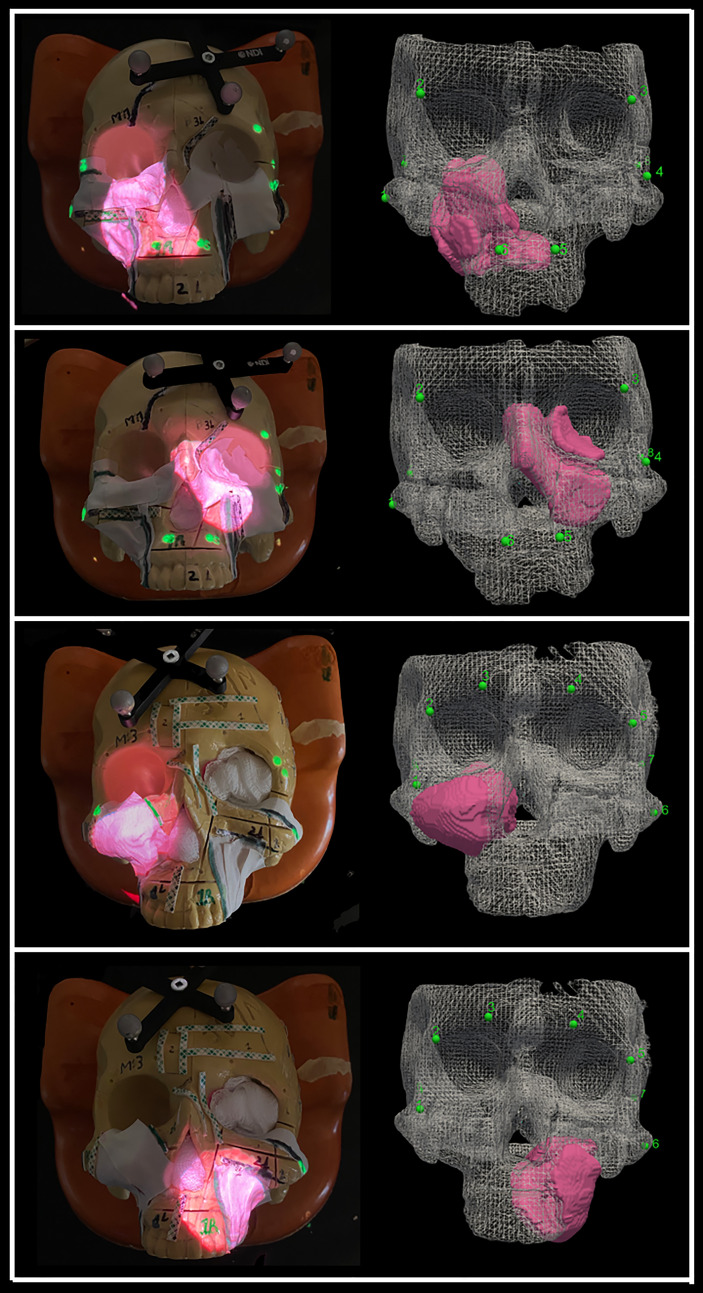
Projection of the four sinonasal tumors using augmented reality, which enables tumor localization. The alignment points are depicted as well as the three-dimensional (3D) reconstruction of the tumors. Pictures were taken without light for demonstration purposes, but good visualization is obtained with light as well.

The AR system was registered into a single coordinate system by pairing correspondent landmarks using fiducial markers identifiable in both the images and projection surface (**Figure 4**). GTx-Eyes provided the 3D surface rendering of the tumors, which were projected by the AR system onto the skulls. The virtual tumor was delineated with CBCT imaging through ITK-SNAP software (ref below). The optical sensor mounted to the projector case facilitated real-time tracking of the AR device to allow the projector and/or skull to be repositioned during tasks without compromising projection accuracy (**Supplementary Video**), with a registration error <1 mm. This approach facilitated identification of the tumors and in consequence guided the virtual cuts of the participants (**Supplementary Figure S1**).

### Gaze-Tracking System

**Figure 5:**
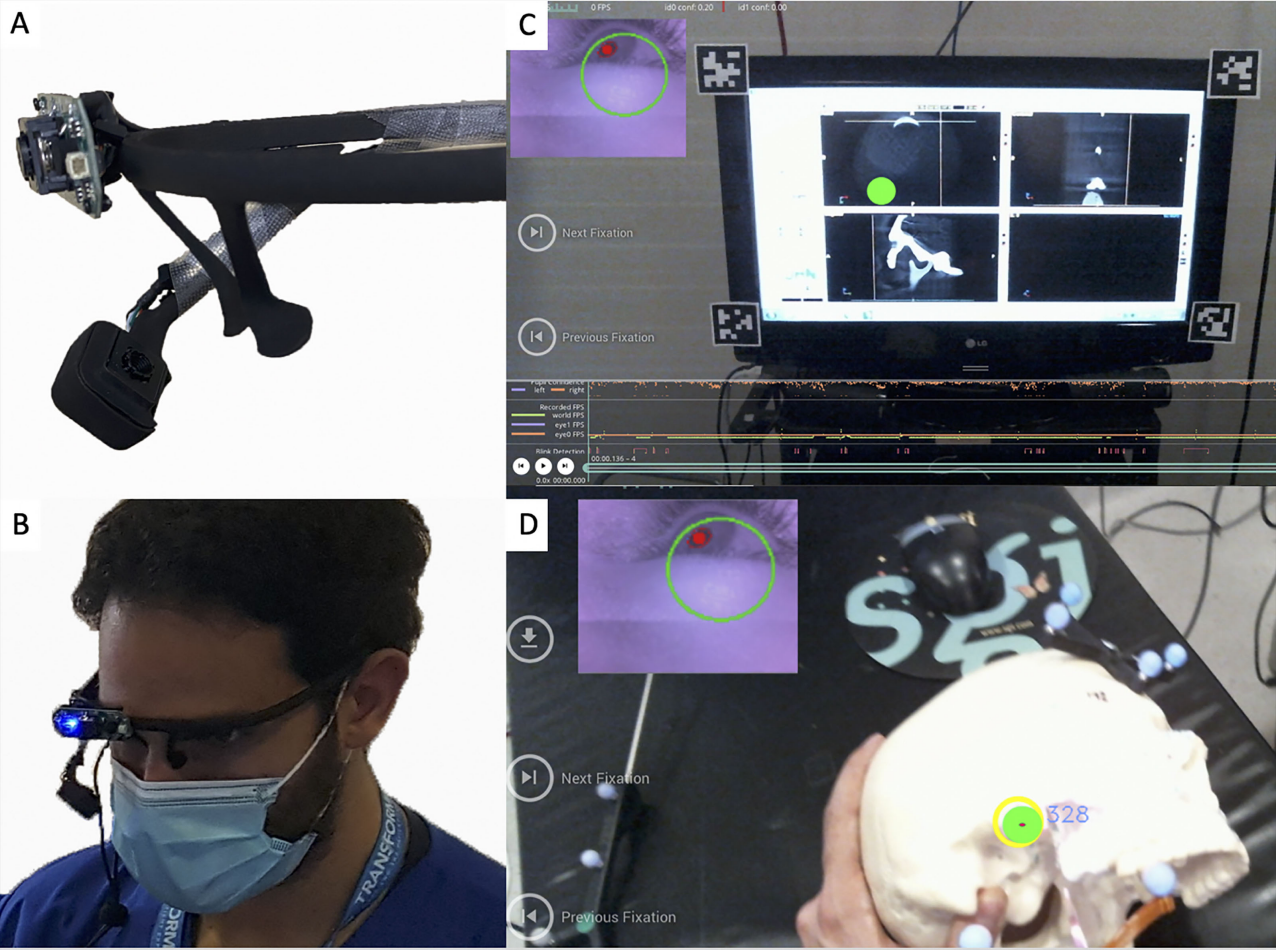
**(A)** Gaze-tracking system with the two cameras that allow it to visualize the pupils and also the participant’s view. **(B)** The device placed on one of the participants. **(C, D)** The “world camera” showing the participant’s view, which could be either on the screen **(C)** or on the surgical field **(D)**. The green dot indicates the exact position of the gaze. A small picture-in-picture screen (upper left) shows the position of the pupil.

A wearable gaze tracker headset was developed to continuously monitor and locate the user’s gaze (e.g., surgical field *vs*. navigation monitor) during surgical tasks. This headset was used solely for gaze-tracking and did not project any information. The eye tracker (Pupil Labs, Berlin, Germany) consists of two cameras: one trained on the eye and the other, a “world camera”, recording the individual’s field of view. A series of computer vision algorithms are applied to the input from the eye camera to reliably detect the pupil throughout the eye’s range of motion. A calibration step provides a triangulating mapping function between the pupil and world cameras, which enables the user’s gaze to be precisely tracked (**Figures 5A–D**).

### Simulations

Five head and neck fellowship-trained surgeons with 3–5 years of experience in oncologic ablations from the Department of Otolaryngology–Head and Neck Surgery of the University Health Network participated in the simulations.

Surgeons were instructed to position the osteotome between the delineated areas of the different osteotomy sites in a sequential order (Pa-FMJ-LIOR-Zy-PMJ) and to provide a 1-cm margin from the tumor along the plane trajectory. Instead of cutting the skulls, virtual cuts were performed in order to allow the reutilization of the models. This involved recording the osteotome position and orientation in the navigation software after the surgeon placed the osteotome in a certain direction and provided confirmation of obtaining the proposed cut. The analysis was performed on the virtual cutting trajectory after all the simulations were completed.

Four procedures were performed: 1) Unguided using axial, sagittal, coronal images; 2) Guided -AR-; 3) Guided -IN-; and 4) Guided -AR and IN-. This last group was possible, as both systems are contained in the same platform software and can be used simultaneously. Analysis of cutting planes was performed using MATLAB software. An area of 4 cm × 2 cm (1 cm on both sides with respect to the longitudinal axis) along the longitudinal axis of the cut was isolated from each plane. The minimal distance with respect to the tumor surface was calculated for each point making up the isolated area and reproduced as a distribution of distances shown as a 4 cm × 2 cm color scaled image. Distance from the tumor surface was classified as “intratumoral” when ≤0 mm, “close” when >0 mm and ≤5 mm, “adequate” when >5 mm and ≤15 mm, and “excessive” when >15 mm. The percentages of points at intratumoral, close, adequate, and excessive distances were calculated for each simulation plane.

The gaze-tracking system was calibrated to each participant, and it was used in all the simulations. The eye-tracking data were analyzed to identify each time point the participants switched their gaze between the navigation monitor and the surgical field. The metric reported is the percentage of total study time spent looking at the navigation monitor.

Finally, a NASA Task Load Index (NASA-TLX) questionnaire that measures the workload of a task was completed by the participants to evaluate the different approaches (18). This questionnaire is widely used and validated and rates the perceived workload to assess a task, system, or other aspects of performance (19–21). It has been employed to assess the workload of new technologies during surgical procedures (22). The total workload is divided into six subjective subscales, which assess mental, physical, and temporal demand, performance, effort, and frustration. Scores range between very low and very high.

### Statistical Analysis

Statistical analysis was run through XLSTAT^®^ (Addinsoft^®^, New York). Simulations were grouped into four categories: unguided, AR, IN, and AR + IN. Rate of intratumoral virtual cuts was the main outcome and was assessed with the Fisher’s exact test. Multivariable analysis adjusting for surgeon and tumor was performed through logistic regression analysis. The groups were also compared in terms of percentage of intratumoral, close, adequate, and excessive distances from the tumor and duration of the simulations through the bilateral Kruskal–Wallis test and Steel–Dwass–Critchlow–Fligner *post-hoc* test. The Kruskal–Wallis test was also employed to analyze the gaze-tracking outcomes and the NASA-TLX scores. Level of significance was set at 0.05 for all statistical tests.

## Results

### Intratumoral Cuts

A total of 335 cuts were simulated. Intratumoral cuts were observed in 20.7%, 9.4%, 1.2%, and 0% of the unguided, AR, IN, and AR + IN simulations, respectively (p < 0.0001). Univariate analysis comparing different procedures with AR showed that this technology improved margins with respect to unguided simulations. The advanced IN approach reduced the intratumoral cut rates compared with AR, and the combination of AR and IN did not significantly decrease the intratumoral cut rate compared with IN alone (p = 0.51). These differences were also seen in a multivariate model adjusted for tumor and surgeon (**Table 1**).

**Table 1:** Intratumoral cuts analysis.

INTRATUMORAL CUTS AND TYPE OF PROCEDURE				
Intratumoral cut/Guidance	Unguided	AR	IN	AR+IN
No	65	77	83	84
Yes	17	8	1	0
Total	82	85	84	84
Percentage	20.7%	9.4%	1.2%	0.0%
**UNIVARIATE ANALYSIS OF INTRATUMORAL CUTS**				
**Guidance**	**OR**	**95%-CI**	**p-value**	
AR	REF	REF	REF	
Unguided	2.44	1.00-5.02	0.049	
SN	0.16	0.03-0.97	0.046	
AR+SN	0.05	0.00-0.97	0.047	
**MULTIVARIATE ANALYSIS OF INTRATUMORAL CUTS**				
**Guidance**	Adjusted* OR	**95%-CI**	**p-value**	
AR	REF	REF	REF	
Unguided	2.54	1.05-6.17	0.039	
SN	0.16	0.03-0.88	0.035	
AR+SN	0.06	0.00-0.81	0.035	

AR Augmented reality, IN intraoperative navigation, OR odds ratio, *Adjusted by tumor model and surgeon.

### Distribution of Points Forming Simulation Planes

The percentage of points forming the simulation planes was also registered. We observed that only the advanced IN system and the combined approach significantly decreased the percentage of intratumoral (p < 0.0001) and close margin points (p = 0.008) compared with the unguided resections (**Supplementary Table S1**).

### Duration of Simulations and Gaze-Tracking Results

Mean total duration of the simulation was 215 s for unguided procedures and 117, 134, and 120 s in the AR, IN, and AR + IN, respectively. Participants required significantly more time to perform the unguided simulations compared to the AR- and IN-guided ones (p = 0.004). There were no differences between the AR-, IN-, and AR + IN-guided procedures. The percentage of time looking at the screen during the procedures was 55.5% for the unguided approaches and 0%, 78.5%, and 61.8% in AR, IN, and AR + IN, respectively (p < 0.001). Adding the AR technology to the combined approach significantly reduced the screen time compared with the advanced IN procedures alone (**Supplementary Table S2**).

### NASA-TLX Scores

We found no differences in scores between the unguided and the AR procedures, and both of them exhibited a high degree of mental demand, effort, and frustration. Combining AR to IN showed a significant improvement on the previous scores (**Supplementary Graph 1** and **Supplementary Table S3**).

## Discussion

In this study, we observed that both advanced IN and AR technologies improved margin delineation compared with unguided procedures. Advanced IN was better for margin delineation than AR but required gaze-toggling between the surgical field and the navigation monitor, whereas AR allowed the surgeon to focus only on the surgical field. The combination of both technologies partially improved the flaws on margins and staring outside the surgical field of the AR and IN techniques, respectively. The integration of AR and IN also improved Mental Demand, Performance, Effort, and Frustration domains in the NASA-TLX questionnaires.

Margin control is among the most important prognostic factors and the only surgeon-controlled variable in head and neck cancer, and efforts have been centered around obtaining clear margins after tumor resections. Nevertheless, positive surgical margins represent a major issue, even in the hand of experienced surgeons. In a report from the largest tertiary referral head and neck cancer center in the Netherlands, 39% out of 69 resections of advanced maxillary tumors (>T3) were incomplete, being posterior and superior margins the most commonly involved (23). In a bi-institutional study from the Cleveland Clinic and the UC San Francisco (24), 24% out of 75 post-maxillectomy patients had positive margins in definitive pathology. Positive margins were associated with a 2-fold increase of risk of death, and in multivariate analysis after controlling for age, nodal stage, and surgical treatment, margins were independently associated with survival (25). Moreover, it has been reported that intraoperative frozen sections (which are probably the only intraoperative resource to evaluate adequacy of the resection) have only 40% sensitivity in open sinonasal approaches (26).

Currently, IN is employed in many centers in endoscopic sinonasal procedures (27–29). By point-tracking an instrument and locating it on two dimensions in triplanar views, IN has shown an increase in accuracy and a reduction in operative time, impacting favorably surgical outcomes and complications. Utilization of IN in open procedures to resect malignant tumors has been less reported, but promising results were obtained in margin status in small cohorts (3, 30, 31). Our group has recently published a preclinical experience utilizing the same advanced IN system used in the present study to assist in open sinonasal approaches (4). The main novelty is that our advanced IN system allows surgeons not only to track the desired instrument but also to visualize the entire cutting trajectory of a tracked cutting tool in 3D. In our previous experience using this technology, eight head and neck surgeons performed 381 simulated osteotomies for the resection of seven tumor models. The use of 3D navigation for margin delineation significantly improved control of margins: unguided cuts had 18.1% intratumoral cuts compared to 0% intratumoral cuts with 3D navigation (p < 0.0001). Furthermore, a clinical study using this advanced IN system for mandibulectomies demonstrated a <1.5-mm accuracy between the planned cuts and the actual bone resection in the post-resection imaging (15). One of the main criticisms to the system by the surgeons in this report was the multitasking challenge between the surgical field and the IN monitor, which can ultimately impact not only efficiency but also patient safety, as the surgeon has to look away from the surgical field.

AR enhances the surgeon’s vision rather than replacing it: CT, PET-CT, or MRI scans can be visualized in 3D and in real time, granting “X-ray vision” to the physician (32, 33). It has recently gained interest by computer-assisted surgery researchers, as it integrates the imaging information onto the surgical field. This has the potential to overcome the main drawback of the IN technology, which stems from the frequent switching of focus from the navigation screens to the surgical field and the translation of 2D imaging data to a 3D anatomical structure (33). Despite having reports on AR application in the field of otolaryngology–head and neck surgery, the majority describe the use of AR using wearable computers (Microsoft HoloLens^®^, Microsoft Corporation, Redmond) and other head-mounted displays (HMDs), which might be cumbersome especially in long procedures, and preclude the use of loupes/headlights. There are literature reports about HMD limitations including heaviness of the devices, breaches in patient privacy/information, battery life, potential lag time secondary to preoperative image processing, and the potential of signal interferences of wireless Internet or Bluetooth connections that may cause intermittent data transmission of image (34). Moreover, most reports of HMD rely solely on the operator visual alignment between the projected images and the anatomical area of interest (35) without any co-registration steps between the projecting surface and the AR system, which can lead to errors. Lastly, there are descriptions of the use of AR in the operating room, but they are merely descriptive and not aimed to improve a surgical task (36) or for educational purposes only (37).

Our study reports several innovations. Tracking the AR projector as well as the projection surface with reflecting markers allowed us to be able to reposition the skull models and the projector without losing accuracy (17, 38), and this is something that was not described previously in head and neck surgery. This is paramount in computer-assisted surgery, as it allows precise projection even when movement occurs, as in real-time situations in the operating room. Another key aspect of our approach is the use of an external projector, which avoids the need for heavy wearable headsets. As a clarification, the headsets used in our study were for gaze-tracking only. The sinonasal/skull base region rigidity represents an excellent indication for AR, as the deformation of tissue is minimal and co-registration is facilitated. Deformation has to be taken into consideration during soft-tissue resections, as it is not possible to adjust the projections during AR (7, 39). By tracking the gaze of the participants, we were able to quantitatively measure the percentage of time that the surgeons had to look away from the surgical field. As our results suggest, there is significant improvement when AR is employed both alone or in combination with IN, addressing the main disadvantages of IN utility. Our AR system shares the same software platform as the advanced IN system, and both approaches can be used concurrently, allowing to evaluate the combination of both. Finally, there is a lack of user evaluation analysis with AR, so we utilized a validated questionnaire to investigate the differences between approaches.

Despite being significantly superior than unguided simulations in terms of intratumoral cut rates, there is room for improvement in our AR system. The advanced IN technology performed better than the AR in terms of intratumoral cut rates, as well as intratumoral and close distribution of points forming the simulation planes. This might be explained by the challenge in finding the correct angle between the projector and the projecting surface. We observed that if the angle differed greatly from 90 degrees, the image can be distorted and therefore lead to inaccuracies in surgical guidance to the operator. For example, when performing the PMJ cuts, by turning the skull 180 degrees, there were cases that the alignment was lost that might have impacted the positive margin cuts. Another important limitation is that the sense of depth can be lost in the projections, and the image can be interpreted in 2D on the surface rather than in 3D, especially with changes in ambient light. One last limitation of image projection is the parallax issue (40). This phenomenon occurs when there is a 3D space non-alignment between the viewer and the projection perspectives. Our system minimizes this issue by adjusting the perspective of the pico-projector close to the surgeon’s sight. In addition, the AR system is fully integrated into our intraoperative navigation system with real-time tracking technology; therefore, the relocation/movement/displacement of the projector will not affect projection accuracy, with no need for further recalibration and registration procedure. These limitations were also reflected on the NASA-TLX scores, where mental demand, effort, and frustration rates for AR were higher than those for IN and similar to those of unguided approaches. Participants commented on the fact that when the adequate angle of projection was lost, they had difficulties interpreting the information from the AR system, which negatively impacted the aforementioned domains of the NASA-TLX questionnaire. A plausible way of improving these flaws, and in consequence improving the margin delineation, is to project the cutting trajectory using AR. Similar to the advanced IN capabilities, the AR could further incorporate the intended cut trajectories on the surgical field, in addition to its projection of the tumor for localization. We also acknowledge the limitations of using preclinical models that may not perfectly replicate the conditions of the operating room. Lastly, another limitation is the non-randomization of the simulated cuts. This was done in order to prevent the participant’s retained memory of the guided views if seen prior to the unguided cuts. Still, the fact that the sequence unguided-AR-IN-AR + IN was followed by all surgeons could have resulted in some degree of learning effect by the participants toward the end of the tasks.

## Conclusion

We reported the use of AR for open sinonasal approaches and improved margin delineation compared with unguided techniques. The advanced IN performed better in terms of margin delineation, but the AR improved the gaze-toggling drawback of IN. Further research within our group is currently underway before translating our experience to clinical practice.

## Data Availability Statement

The original contributions presented in the study are included in the article/**Supplementary Material**. Further inquiries can be directed to the corresponding author.

## Ethics Statement

Ethical review and approval was not required for the study on human participants in accordance with the local legislation and institutional requirements.

## Author Contributions

All authors listed have made a substantial, direct, and intellectual contribution to the work and approved it for publication.

## Funding

This is a self-funded study. Funding was provided by the Princess Margaret Cancer Foundation (Toronto, Canada), including the Kevin and Sandra Sullivan Chair in Surgical Oncology, the Myron and Berna Garron Fund, the Strobele Family Fund, and the RACH Fund.

## Conflict of Interest

The authors declare that the research was conducted in the absence of any commercial or financial relationships that could be construed as a potential conflict of interest.

## Publisher’s Note

All claims expressed in this article are solely those of the authors and do not necessarily represent those of their affiliated organizations, or those of the publisher, the editors and the reviewers. Any product that may be evaluated in this article, or claim that may be made by its manufacturer, is not guaranteed or endorsed by the publisher.

## Supplementary Material

Supplementary Video Advanced Intraoperative Navigation system showing the entire cutting trajectory with respect to the tumor, and the Augmented Reality system providing the exact location of the tumor. The co-registration of the skull and the projector in the Augmented Reality system allows the image to be accurately projected and automatically adjusted despite movements.
