# Supplementary material for: Augmented Reality and Intraoperative Navigation in Sinonasal Malignancies: A Preclinical Study
Source: Front Oncol. 2021 Nov 1;11:723509. doi: 10.3389/fonc.2021.723509 (PMC8591179; doi:10.3389/fonc.2021.723509)
Supplement: Supplementary file 2 [file DataSheet_1.docx]

**Supplementary Video**. Advanced Intraoperative Navigation system showing the entire cutting

trajectory with respect to the tumor, and the Augmented Reality system providing the exact

location of the tumor. The co-registration of the skull and the projector in the Augmented

Reality system allows the image to be accurately projected and automatically adjusted despite

movements.

**Supplementary Figure 1:** Panels depicting the virtual cuts using the Augmented Reality technology for two maxillary tumors.


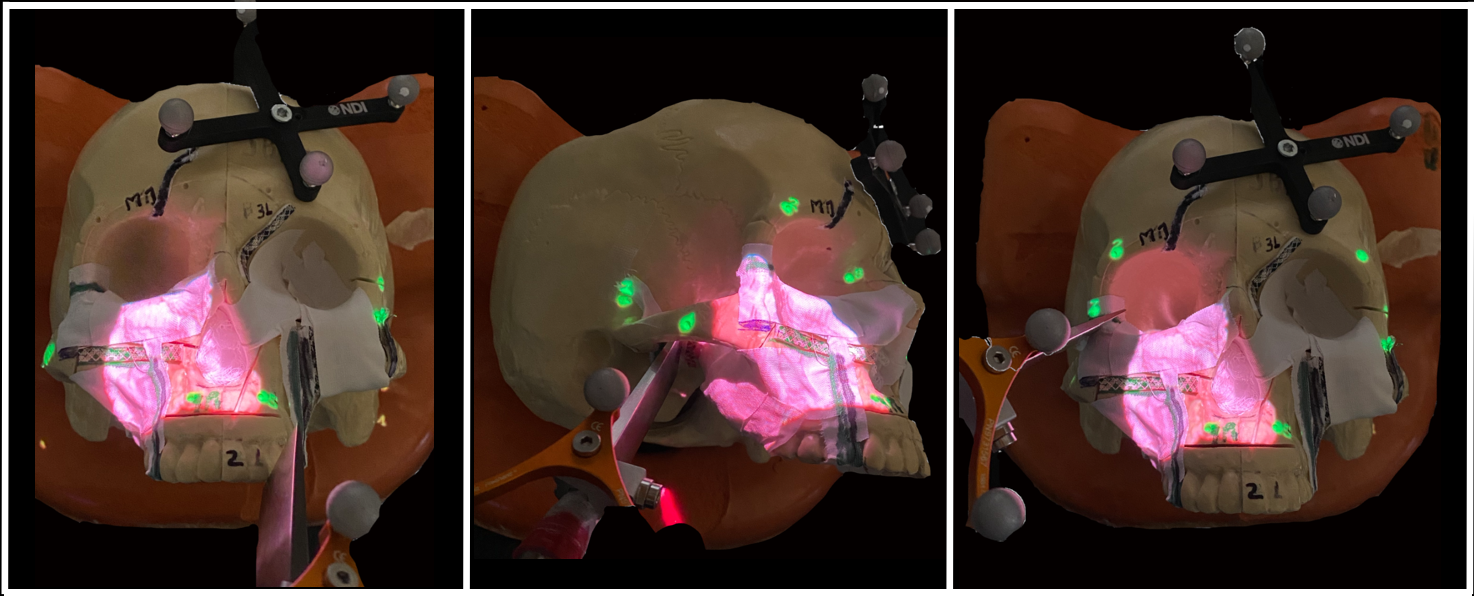

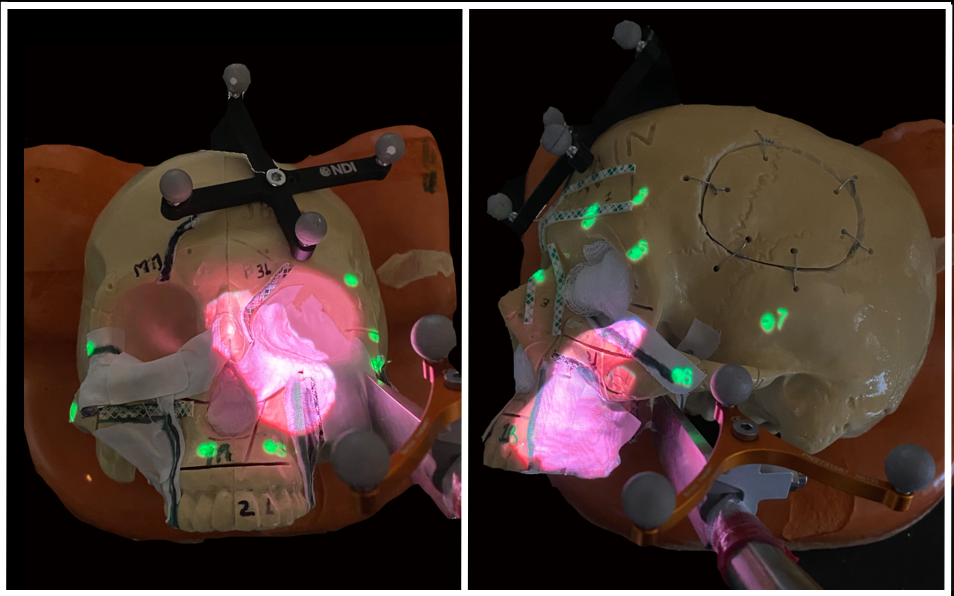


**Supplementary Table 1:** Distribution of points forming simulation planes.

| **Outcome** | **Unguided** | **AR** | **IN** | **AR+IN** | **p-value (Kruskal Wallis test)** |
| --- | --- | --- | --- | --- | --- |
| Intratumoral points (mean [IQR]) | 3.0%  [0.0-0.0%]^A^ | 1.4%  [0.0-0.0%]^A^ | 0.0%  [0.0-0.0%]^B^ | 0.0%  [0.0-0.0%]^B^ | ***<0.0001*** |
| Close points (mean [IQR]) | 11.7%  [0.0-22.9%]^A^ | 7.6%  [0.0-13.7%]^A,B^ | 3.7%  [0.0-4.6%]^B^ | 3.8%  [0.0-3.0%]^B^ | ***0.008*** |
| Excessive points (mean [IQR]) | 29.1%  [3.5-42-2%]^A^ | 28.9%  [7.7-46.8%]^A^ | 30.9%  [5.9-48.5%]^A^ | 29.2%  [8.5-44.3%]^A^ | 0.865 |
| Adequate points (mean [IQR]) | 56.2%  [42.3-72.0%]^A^ | 62.1%  [39.6-83.2%]^A,B^ | 65.4%  [51.5-85.7%]^A,B^ | 67.0%  [49.6-87.7%]^B^ | ***0.021*** |

^A,B^Groups significantly differed based on Steel-Dwass-Critchlow-Filgner post-hoc test. IQR- Interquartile range.

*AR Augmented reality, IN intraoperative navigation.*

**Supplementary Table 2**: Total duration and screen time during the simulations.

| **Outcome** | **Unguided** | **AR** | **IN** | **AR+IN** | **p-value (Kruskal Wallis test)** |
| --- | --- | --- | --- | --- | --- |
| Total duration  (mean [IQR]) | 215.8”  [154.7-263.8”]^A^ | 117.4”  [83.4-145.0”]^B^ | 134.7”  [83.7”-170”]^B^ | 120.9”  [81.8-145.0”]^B^ | ***0.0004*** |
| Screen time (mean [IQR]) | 55.5%  [51.3-59.0%]^A^ | 0.0%  [0.0-0.0%]^B^ | 78.5%  [72.6-83.9%]^C^ | 61.8%  [53.4-70.0%]^A^ | ***<0.0001*** |

^A,B,C^Groups significantly differed based on Steel-Dwass-Critchlow-Filgner post-hoc test. IQR- Interquartile range.

*AR Augmented reality, IN intraoperative navigation.*

**Supplementary Graph 1:** NASA-TLX median values.


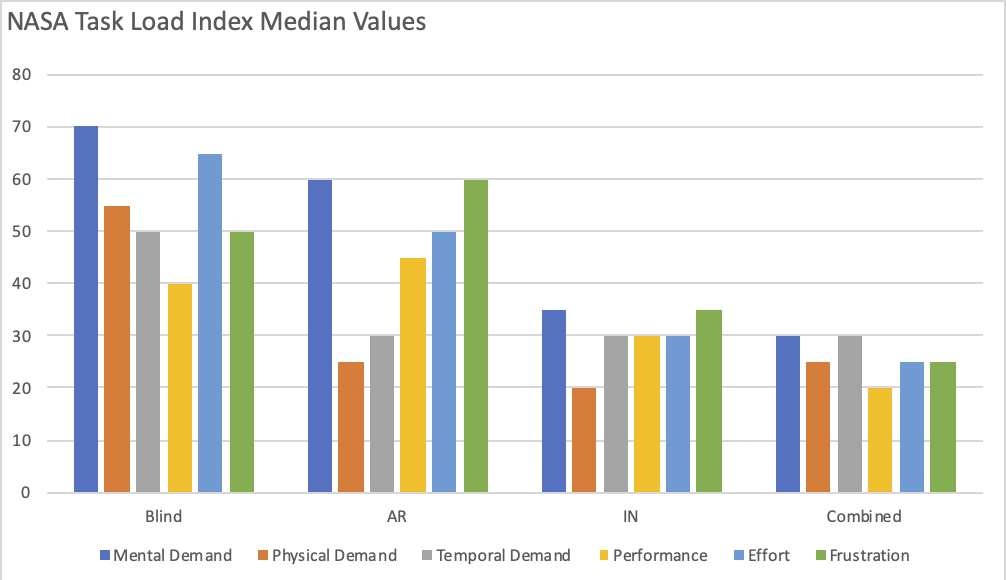


**Supplementary Table 3**: NASA-TLX scores analysis.

| **Outcome** | **Unguided** | **AR** | **IN** | **AR+IN** | **p-value (Kruskal Wallis test)** |
| --- | --- | --- | --- | --- | --- |
| Mental demand  (median) | 70^A^ | 60^A,B^ | 35^B^ | 30^B^ | ***0.002*** |
| Physical demand (median) | 55^A^ | 25^A^ | 20^A^ | 25^A^ | 0.474 |
| Temporal demand (median) | 50^A^ | 30^A^ | 30^A^ | 30^A^ | 0.516 |
| Performance (median) | 40^A^ | 45^A^ | 30^A,B^ | 20^B^ | ***0.006*** |
| Effort (median) | 65^A^ | 50^A^ | 30^B^ | 25^B^ | ***0.002*** |
| Frustration (median) | 50^A^ | 60^A^ | 35^A,B^ | 25^B^ | ***0.010*** |

^A,B^Groups significantly differed based on Steel-Dwass-Critchlow-Filgner post-hoc test. IQR - Interquartile range.

*AR Augmented reality, IN intraoperative navigation.*
